# Differences in food sensitization patterns between infants/toddlers and children: a cross-sectional study of patients aged 0–14 years in urban China

**DOI:** 10.3389/falgy.2026.1802814

**Published:** 2026-05-20

**Authors:** Huimin Huang, Haisheng Hu, Linshu Xu, Yanting Fang, Yinghong Lin, Na Li, Baoqing Sun

**Affiliations:** 1 Department of Clinical Laboratory, State Key Laboratory of Respiratory Disease, National Center for Respiratory Medicine, National Clinical Research Center for Respiratory Disease, Guangzhou Institute of Respiratory Health, The First Affiliated Hospital of Guangzhou Medical University, Guangzhou, China; 2 Guangzhou National Laboratory, Guangzhou, China; 3 Guangdong Provincial Clinical Research Center for Laboratory Medicine, Guangzhou, China; 4 Children’s Neurorehabilitation Laboratory, Shenyang Children’s Hospital, Shenyang, China

**Keywords:** allergen, childhood allergies, epidemiology, food sensitization, sIgE

## Abstract

**Objective:**

With rapid urbanization in China, the prevalence of food allergies among children has significantly increased. To explore the evolution of sensitization patterns in early life, this study aimed to systematically compare allergen profiles among infants/toddlers and children with food sensitization to provide evidence-based support for implementation.

**Method:**

This study analyzed sensitization to 7 common food allergens in 947 patients aged 0–14 years (402 children aged 4–14 years and 545 infants/toddlers aged 0–3 years) with food sensitization in urban of China.

**Results:**

In children, spring and autumn visits were the more common (all at 26.4%), and the highest sensitization rate was egg whites (49.0%), followed by hazelnuts (43.8%), and milk (32.6%). In infants/toddlers, winter visits were the most common (28.3%), and the highest sensitization rate was egg white (75.4%), followed by milk (55.4%) and wheat (15.2%). Meanwhile, the positive rates for egg whites (75.4% vs. 49.0%, *P* < 0.001) and milk (55.4% vs. 32.6%, *P* < 0.05) were significantly higher in infants/toddlers than in children. Positive rates of hazelnut (43.8% vs. 11.6%, *P* < 0.001) were significantly higher in children than in infants/toddlers. The distribution of different allergens also varies among infants/toddlers, and children during different seasons. Infants/toddlers with skin symptoms had the highest positivity rates for shrimp (46.9%) and beef (45.6%), whereas children had the highest positivity rates for codfish (27.3%) and shrimp (22.4%). Compared with children (34.1%), >44.6% of infants/toddlers were co-sensitized to ≥2 allergens. Optimal scale analysis revealed no fixed pattern of sensitization among infants/toddlers. If it was sensitization to one of the allergens, it was highly likely to be co-sensitized to others (Cronbach's α = 0.924). However, in children, one group was primarily sensitized to egg whites, codfish, and shrimp, whereas the other was primarily to milk, wheat, and hazelnuts (Cronbach's α = 0.876).

**Conclusion:**

Children of different ages showed distinct sensitization characteristics, and the sensitization pattern of children may have developed from that of infancy. These findings support the adoption of differentiated allergy risk management and prevention strategies at different life stages.

## 1 Introduction

With the acceleration of urbanization in China, the prevalence of childhood food allergies, a major public health concern, has significantly increased. The onset and progression of allergic reactions are strongly age-dependent (1, 2). The period from infancy to early childhood constitutes a critical window for immune maturation and the establishment of tolerance to external antigens, however, it also represents a phase of heightened vulnerability to primary sensitization against dietary proteins (3). During this stage, immune reactivity to novel protein antigens remains labile, predisposing individuals to initial sensitization. As children grow, the progressive maturation of immune regulatory mechanisms coupled with expanding dietary diversity drives the dynamic evolution of sensitization profiles (4). Food proteins that trigger allergic responses in early life (e.g., milk and eggs) may later become clinically tolerated, whereas sensitization risk may shift toward newly introduced food antigens (e.g., tree nuts and seafood) (5). Supporting this, a recent Australian cohort study found that milk and egg sensitization predominated at age ≤ 1, while the prevalence of peanut and tree nut sensitization increased by ages 4–6 (6). This highlights the complex interplay between developmentally programmed immune pathways and cumulative antigen exposure during childhood (7).

Therefore, understanding the differences in sensitization characteristics and patterns of evolution between infants/toddlers and children is essential for developing targeted strategies for prevention, diagnosis, and treatment (8). However, data on the characteristics of food sensitization between infants/toddlers and children in the urban areas of China remain lacking, especially after the COVID-19 epidemic. To address this knowledge gap, this study aimed to analyze the data from children aged 0–14 years with food sensitization in urban China, comparing infants/toddlers (0–3 years) and children (4–14 years) in terms of sensitization patterns to common food allergens, elucidated the age-related evolution of food sensitization characteristics and provide a scientific basis for early clinical intervention and individualized management.

## 2 Method

### 2.1 Study design

This was a cross-sectional study. The study included 947 pediatric patients with food sensitization who underwent serum food allergen-specific immunoglobulin E (sIgE) determination using magnetic particle luminescence technology (ALLEOS 2000, China) from January 1, 2024, to June 30, 2025. The patients, aged 0–14 years, from urban areas of China, were collected by The First Affiliated Hospital of Guangzhou Medical University and Shenyang Children's Hospital. Patients with cancer, immunodeficiency, parasitic infections, or autoimmune diseases were also excluded. All patients immediately sought medical attention due to respiratory discomfort, skin redness, itching, gastrointestinal discomfort, and other typical allergic symptoms after eating. To identify the causative allergens (s), serum sIgE levels were measured using a panel of seven food allergens: egg white (F1), milk (F2), codfish (F3), wheat (F4), hazelnuts (F17), shrimp (F24), and beef (F27). Only patients who tested positive for at least one food allergen (sIgE) were included in the analysis and divided into an infant/toddler group (0–3 years old) and a children group (4–14 years old).

### 2.2 Detection

Serum samples were collected from the hospital blood collection room using separation gel vacuum coagulation tubes. A venous blood sample (5 mL was drawn and centrifuged for 10 min at 3,000 r/min. Sera allergen sIgE were analyzed using magnetic particle luminescence technology (ALLEOS 2000, China) by trained technicians, and the results were reported in kU/L, with ≥0.35 kU/L as a positive cutoff. Based on the quantitative sIgE levels, the reactivity was categorized into six classes: Class 1 (≥0.35 kU/L to <0.70 kU/L), Class 2 (≥0.70 kU/L to <3.50 kU/L), Class 3 (≥3.50 kU/L to <17.50 kU/L), Class 4 (≥17.50 kU/L to <50.00 kU/L), Class 5 (≥50.00 kU/L to <100.00 kU/L), and Class 6 (≥100.00 kU/L).

### 2.3 Statistical method

Data were analyzed using SPSS software (version 22.0; IBM Corp., Armonk, NY, USA). Normally distributed data, such as age, are presented as mean ± standard deviation. Qualitative information, such as the positivity rate, is presented as a percentage or frequency. Inter-group difference was assessed using the chi-square test (*χ*^2^). The interrelationships among allergens were analyzed using optimal scale analysis for classification, and statistical significance was defined as *P* < 0.05.

## 3 Result

### 3.1 Differences in sensitization situation between children and infants/toddlers

**Figure 1:**
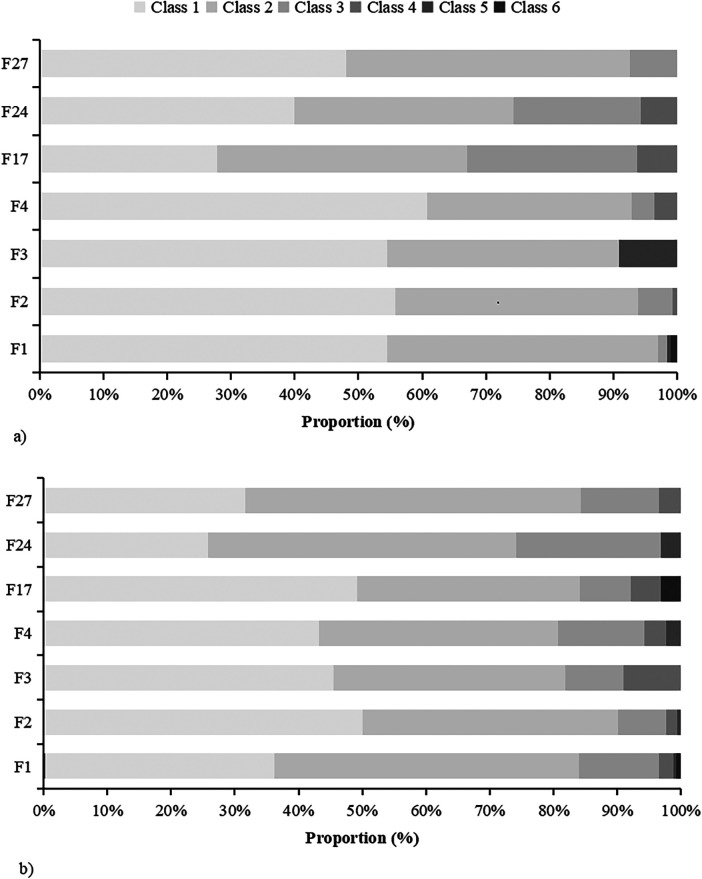
Differences in sensitization levels between the children and infant/toddler groups. The Class of different allergens between **(a)** children and **(b)** infants/toddlers with food allergies. F1: egg white; F2: milk; F3: codfish; F4: wheat; F17: hazelnut; F24: shrimp and F27: beef.

In total, 402 children and 545 infants/toddlers were included in this study. In children, 61.4% were male, the average age was 6.64 ± 2.29 years, 53.2% had respiratory symptoms, and 7.2% had digestive tract symptoms. Spring and autumn visits were the more common (all at 26.4%). The highest sensitization rate was for egg whites (49.0%), followed by hazelnuts (43.8%), milk (32.6%), and wheat (13.7%). In infant/toddler patients, 54.5% were male, with an average age of 2.08 ± 1.10 years. Among them, 35.6% had respiratory symptoms, and 12.1% had digestive tract symptoms. Winter visits were the most common (28.3%). The highest sensitization rates were observed for egg whites (75.4%), followed by milk (55.4%), wheat (15.2%), and hazelnut (11.6%). In addition, the positive rates for egg whites (75.4% vs. 49.0%, *χ*^2^ = 10.98, *P* < 0.001) and milk allergens (55.4% vs. 32.6%, *χ*^2^ = 7.41, *P* = 0.032) were significantly higher in infants/toddlers than in children. However, the positive rates for hazelnut allergens (43.8% vs. 11.6%, *χ*^2^ = 53.51, *P* < 0.001) were significantly higher in children than in infants/toddlers (Table 1). In infants/toddlers and children, the class of food sensitization in >80% of patients was low (Classes 1–3) (Figure 1).

**Table 1:** Baseline differences between the children and infant/toddler groups.

Characteristic	Infant/toddler	Children	*P*
Total (*N*)	545	402	–
Age (years)	2.08 ± 1.10	6.64 ± 2.29	<0.001
Gender (male/female, *n*)	297/248	247/155	0.076
Symptom (*n*, %)			
Respiratory	194, 35.6%	214, 53.2%	<0.001
Skin	166, 30.5%	65, 16.2%	
Digestive tract	66, 12.1%	29, 7.2%	
Other	119, 21.8%	94, 23.4%	
Season (*n*, %)			
Spring	146, 26.8%	106, 26.4%	0.085
Summer	140, 25.7%	92, 22.9%	
Autumn	105, 19.3%	106, 26.4%	
Winter	154, 28.3%	98, 24.4%	
Positive for allergen (*n*, %)			
F1	411, 75.4%	197, 49.0%	<0.001
F2	302, 55.4%	131, 32.6%	0.032
F3	22, 4.0%	22, 5.5%	0.064
F4	83, 15.2%	55, 13.7%	0.092
F17	63, 11.6%	176, 43.8%	<0.001
F24	31, 5.7%	35, 8.7%	0.074
F27	57, 10.5%	27, 6.7%	0.081

*P* value was calculated using the chi-square test. F1, egg white; F2, milk; F3, codfish; F4, wheat; F17, hazelnut; F24, shrimp; F27, beef. Other symptoms included eye redness, itching, tearing, oral allergy syndrome, and throat discomfort.

### 3.2 Differences in sensitization between children and infants/toddlers in different seasons

**Figure 2:**
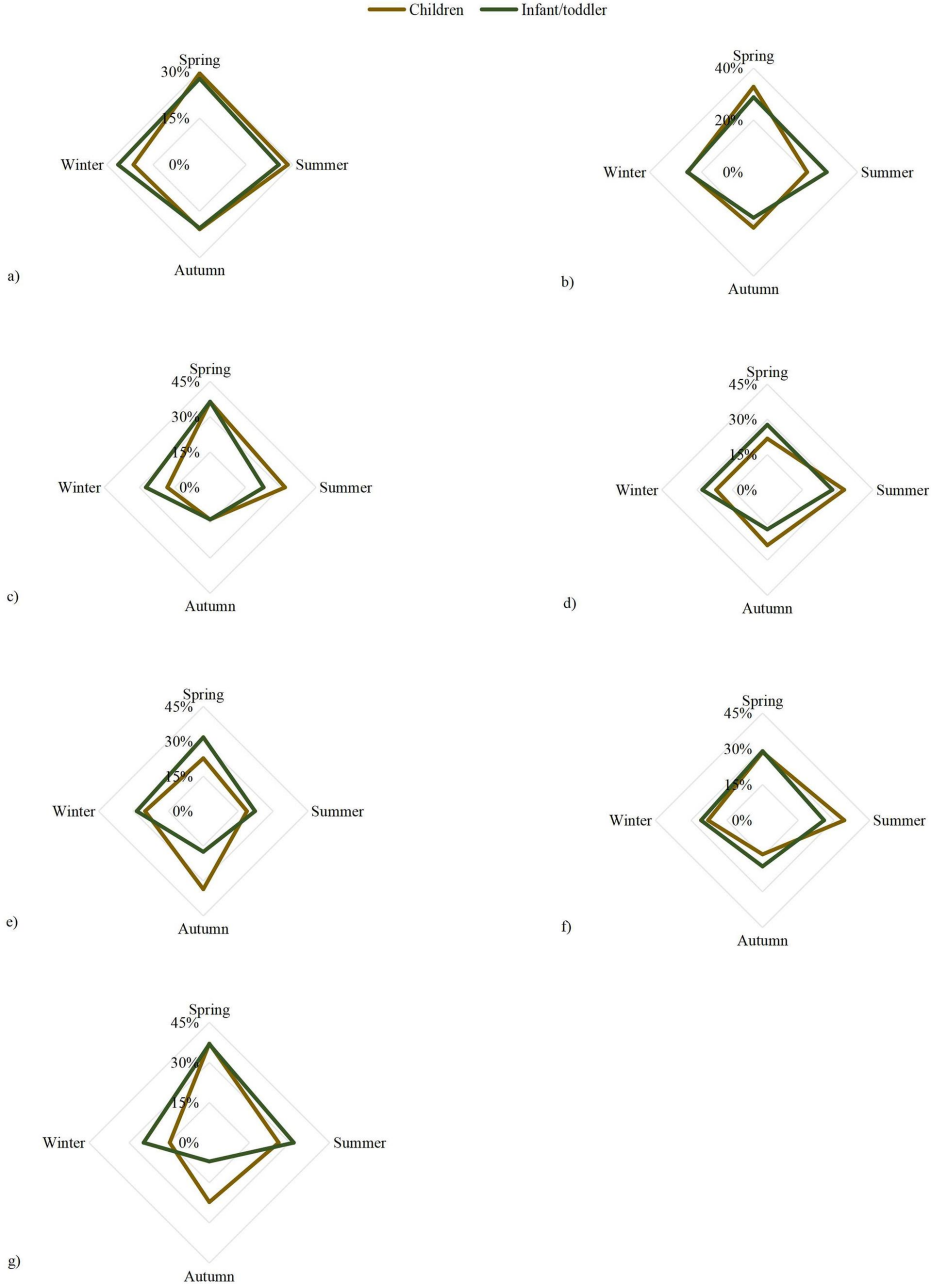
Differences in sensitization between the children and infant/toddler groups in different seasons differences in **(a)** egg white, **(b)** milk, **(c)** codfish, **(d)** wheat, **(e)** hazelnut, **(f)** shrimp, and **(g)** beef allergens between infants/toddlers and children with food sensitization in different seasons.

The sensitization patterns of common food allergens exhibited distinct seasonal variations between children and infants/toddlers. As shown in Figures 2a–c, sensitization to egg white, milk, and codfish peaked in spring for both groups. In contrast, seasonal peaks differed for other allergens: wheat sensitization was most common in summer among children but in spring among infants/toddlers (Figure 2d). Similarly, hazelnut sensitization peaked in autumn for children and in spring for infants/toddlers (Figure 2e), while shrimp sensitization was highest in summer for children and in spring for infants/toddlers (Figure 2f). Sensitization to beef peaked in spring in both groups (Figure 2g).

### 3.3 Differences in sensitization between children and infants/toddlers with different symptoms

In infants/toddlers, patients with respiratory symptoms had the highest positivity rates for codfish (45.5%) and hazelnuts (46.5%), whereas patients with skin symptoms had the highest positivity rates for shrimp (46.9%) and beef (45.6%). Children with respiratory symptoms had the highest positivity rates for beef (66.7%) and milk (59.5%), whereas patients with skin symptoms had the highest positivity rates for codfish (27.3%) and shrimp (22.4%). In addition, for respiratory symptoms, positive rates of egg white (53.8% vs. 33.6%, *χ*^2^ = 4.31, *P* = 0.038), milk (59.5% vs. 36.8%, *χ*^2^ = 17.12, *P* < 0.001), shrimp (47.8% vs. 18.8%, *χ*^2^ = 8.26, *P* = 0.016) and beef (66.7% vs. 26.3%, *χ*^2^ = 17.53, *P* = 0.029) in children were significantly higher than in infants/toddlers. For skin symptoms, positive rates of egg white (32.1% vs. 17.3%, *χ*^2^ = 52.07, *P* < 0.001), wheat (38.3% vs. 13.1%, *χ*^2^ = 25.42, *P* = 0.024), shrimp (46.9% vs. 22.4%, *χ*^2^ = 14.67, *P* = 0.006) and beef (45.6% vs. 14.8%, *χ*^2^ = 39.14, *P* = 0.029) in infants/toddlers were significantly higher than in children (Table 2).

**Table 2:** Positivity rates of food allergens in the children and infant/toddler groups with different symptoms.

Positivity rate (*n*, %)	Infant/toddler				Children			
	Respiratory	Skin	Digestive tract	Other	Respiratory	Skin	Digestive tract	Other
F1	138, 33.6%	132, 32.1%	44, 10.7%	97, 23.6%	106, 53.8%	34, 17.3%	18, 9.1%	39, 19.8%
F2	111, 36.8%	89, 29.5%	40, 13.2%	62, 20.5%	78, 59.5%	18, 13.7%	9, 6.9%	26, 19.8%
F3	10, 45.5%	7, 31.8%	4, 18.2%	1, 4.5%	13, 59.1%	6, 27.3%	1, 4.5%	2, 9.1%
F4	28, 32.1%	30, 38.3%	4, 4.3%	21, 25.3%	25, 42.9%	9, 13.1%	8, 17.9%	13, 26.2%
F17	29, 46.5%	19, 33.3%	3, 5.3%	12, 14.9%	89, 47.8%	30, 16.7%	11, 7.5%	46, 28.0%
F24	8, 18.8%	12, 46.9%	2, 7.8%	9, 26.6%	17, 47.8%	6, 22.4%	2, 7.5%	10, 22.4%
F27	15, 26.3%	26, 45.6%	4, 7.0%	12, 21.1%	18, 66.7%	4, 14.8%	2, 7.4%	3, 11.1%

F1, egg white; F2, milk; F3, codfish; F4, wheat; F17, hazelnut; F24, shrimp; F27, beef.

### 3.4 Differences in co-sensitization patterns between children and infants/toddlers

**Figure 3:**
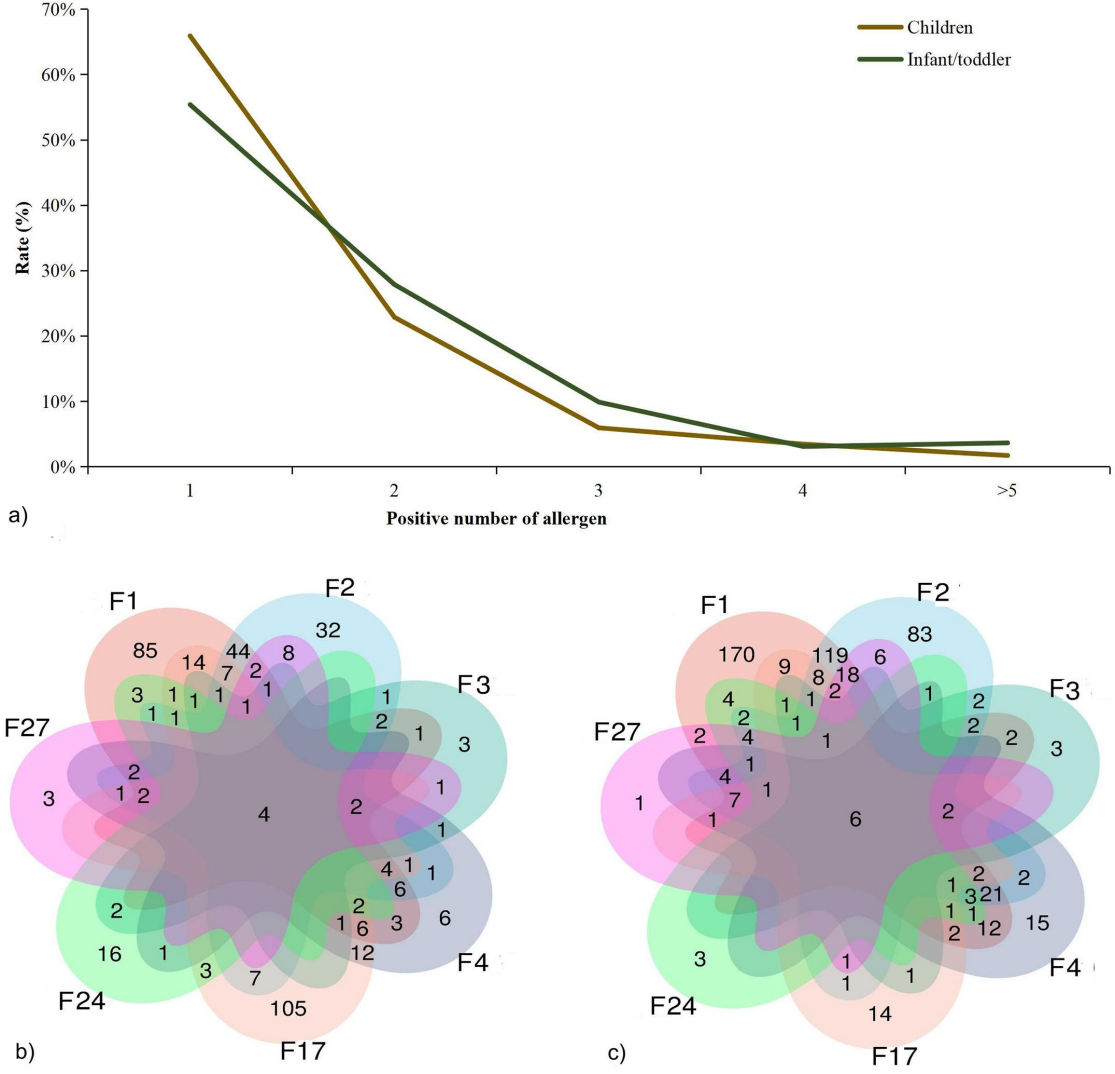
Differences in multiple sensitization between the children and infant/toddler groups. **(a)** The number of co-positive allergens in patients and Wayne diagram analysis of co-sensitization between **(b)** infants/toddlers, and **(c)** children. F1: egg white; F2: milk; F3: codfish; F4: wheat; F17: hazelnut; F24: shrimp and F27: beef.

**Figure 4:**
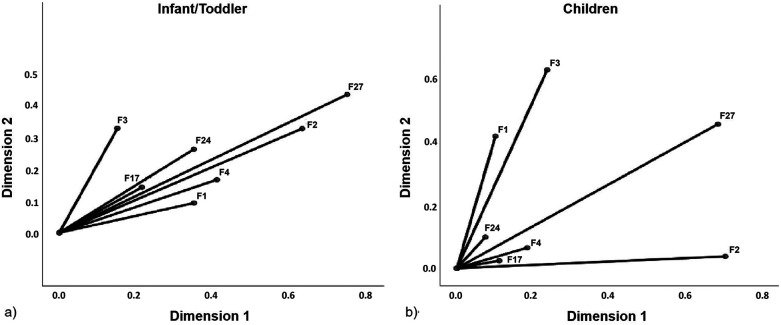
Differences in sensitization patterns between the children and infant/toddler groups. Optimal scale analysis for classification of sensitization patterns in **(a)** infants/toddlers and **(b)** children. F1: egg white; F2: milk; F3: codfish; F4: wheat; F17: hazelnut; F24: shrimp and F27: beef.

For co-sensitization, compared to children (34.1%), >44.6% of infants/toddlers were positive for ≥2 allergens (Figure 3a) and many tested positive for both egg whites and milk simultaneously (Figure 3b). Among children, the highest number of patients were positive for hazelnuts alone, followed by egg whites (Figure 3c). Interestingly, optimal scale analysis showed that co-sensitization among allergens was closely related in infants/toddlers, suggesting no fixed pattern of sensitization. If it is sensitization to one of the allergens, it was highly likely to be co-sensitized to other allergens (Cronbach's α = 0.924) (Figure 4a). However, they can be divided into two sensitization groups: one primarily composed of egg white, codfish, and shrimp, while the other group of milk, wheat, and hazelnut in children (Cronbach's α = 0.876) (Figure 4b).

## 4 Discussion

A comparison of food sensitization characteristics in infants/toddlers and children provides valuable insights into the age-related evolution of sensitization. In this cross-sectional study conducted in urban China, we systematically compared two groups and identified distinct differences in allergen profiles, seasonal distributions, clinical symptoms, and co-sensitization patterns. Egg white was the most common allergen in both groups, with a significantly higher sensitization rate in infants/toddlers. This finding is consistent with that of a prospective Australian study reporting egg white as a key early allergen, with most cases resolving spontaneously in early childhood (9). This natural resolution likely explains the higher prevalence in younger children and implies that interventions during infancy could modulate the sensitization process (10–12). Supporting this, studies conducted in the United States indicate that delaying egg introduction (>12 months) significantly increases the risk of egg sensitization at 2 and 12 years of age (13). Therefore, the appropriate introduction of eggs during infancy (≤12 months) is recommended to mitigate the risk of egg sensitization later in life. Similarly, the milk sensitization rate was significantly higher in infants/toddlers, a pattern attributable to their early and predominant exposure to dairy-based diets. This homogeneous dietary exposure may increase the risk of sensitization, underscoring the need for complementary preventive strategies (14). Emerging evidence suggests that modulating the early-life environment through nutritional interventions can influence the development of allergies. For example, a specific probiotic combination (*Lactobacillus rhamnosus* and *Lactobacillus casei*) has been shown to significantly improve skin symptoms in children under 2 years of age with atopic dermatitis and cow's milk protein sensitization, particularly in those already sensitized (15). Furthermore, Ito et al. indicated that daily intake of a small amount of regular cow's milk formula in infants aged 1 and 2 months can significantly reduce the risk of cow's milk protein sensitization (16). Collectively, these findings highlight that early and targeted nutritional strategies may help shape the sensitization trajectory and alter the natural history of allergies.

Regarding seasonal variations, spring exhibited the highest sensitization rate for most allergens. This pattern may be linked to elevated pollen concentrations during spring, potentially triggering cross-reactions, especially with plant-derived allergens, such as wheat and hazelnuts (17). Lipid transfer protein (LTP)-mediated cross-reactivity plays a prominent role in plant-food cross-sensitization (18). Hazelnut, for instance, is a core food in LTP syndrome, with Cor a 8 as its major LTP allergen, whereas wheat contains Tri a 14, another significant LTP allergen (19). Further research is warranted to clarify the clinical and immunological relevance of seasonal and molecular associations.

We also observed specific associations between the clinical symptoms and allergens. Sensitization to shrimp and beef was more prominent among infants/toddlers with skin symptoms, whereas sensitization to shrimp and codfish was more prominent among children. The major allergen in crustaceans such as shrimp is tropomyosin, a heat- and digestion-resistant protein that can readily cross the intestinal barrier and induce IgE-mediated reactions, often presenting as urticaria, eczema exacerbation, or angioedema (20, 21). The skin barrier function in infants and children is not yet fully developed, which may make it easier for allergens to penetrate or induce pronounced inflammatory reactions. Thus, children presenting with cutaneous symptoms should be evaluated for shrimp and fish sensitization, and dietary guidance should be considered to reduce exposure.

Notably, infants/toddlers demonstrated a higher propensity for co-sensitization, with nearly half testing positive for ≥2 allergens, and strong correlations were observed among them, indicating a lack of fixed sensitization patterns. This likely reflects immune system immaturity (a shift in the Th1/Th2 balance and impaired intestinal barrier function), leading to a broad tendency toward sensitization to multiple food proteins. In contrast, children exhibited a more distinct clustering of sensitization, characterized by a seafood-dominated group (primarily egg whites, codfish, and shrimp) and a land-food-dominated group (primarily milk, wheat, and hazelnuts). This suggests that as the immune system matures and dietary exposure diversifies, allergic responses become more antigen-specific. Recent studies have provided mechanistic evidence of these patterns. The clustering of sensitization to high-quality animal proteins (e.g., eggs and seafood) may stem from shared exposure, whereas genetic factors, such as filaggrin (FLG) mutations, identified in Asian and European birth cohorts as potential biomarkers for persistent egg sensitization, may also contribute (16). Moreover, children with milk sensitization often exhibit an underlying atopic constitution that predisposes them to broader apoty tendencies, including pollen sensitization (22). For those at high risk, early dietary intervention using a specific partially hydrolyzed whey protein formula reduces the incidence of atopic diseases within the first 6 months of life.

### 4.1 Limitations and future directions

This study had some limitations. First, although all participants were uniformly tested for the same panel of seven common food allergens, thus providing consistent data for comparing multi-sensitization patterns, the sample size might have limited the generalizability of some subgroup findings. Meanwhile, considering the age and clinical feasibility, the food challenge test was not performed, but this is also a limitation of the real medical world. Second, the allergen panel did not include all potentially sensitizing food proteins, we only test 1–2 important allergen in same sensitization types simultaneously, so it could not rule out sensitization to other allergens that were not tested, such as peanuts, soybeans, sesame seeds, etc. Third, the cross-sectional design precluded long-term follow-up to observe the natural evolution of sensitization profiles. Future prospective cohort studies incorporating expanded allergen detection, and detailed environmental exposure data are needed to elucidate the mechanisms and developmental trajectories of food sensitization in children. In addition, further research is needed to obtain more accurate clinical relevance of childhood allergies, such as the association between respiratory symptoms and food allergen.

## 5 Conclusion

The results of this study show that infants/toddlers and children exhibit different sensitization characteristics. Egg whites had the highest sensitization rate in both groups, especially in infants/toddlers. Meanwhile, infants/toddlers did not show a fixed sensitization pattern, whereas children showed two types: one dominated by egg white and the other by milk, the sensitization pattern of children may develop from that of infancy. The findings of this study support the adoption of differentiated allergy risk management and prevention strategies at different life stages.

## Data Availability

The raw data supporting the conclusions of this article will be made available by the authors, without undue reservation.
